# Practical issues of biomarker-assisted targeted therapy in precision medicine and immuno-oncology era

**DOI:** 10.1136/esmoopen-2018-000370

**Published:** 2018-06-12

**Authors:** Dae Ho Lee

**Affiliations:** Department of Oncology, University of Ulsan College of Medicine, Asan Medical Center, Songpa-gu, Seoul, Republic of Korea

**Keywords:** immuno-oncology

## Abstract

The concept of precision medicine is not new, as multiplex and very sensitive methods, or next-generation sequencing and matched targeted cancer therapies, have come to clinical practice. Substantial progress has been made from the discovery to the development and clinical application of biomarkers and matched targeted therapies. However, there still remain many challenges and issues to be overcome in each step, from acquisition of tumour tissues through validation of biomarkers to the final decision on targeted therapy. This review will briefly touch on these issues, hoping to provide a better understanding and application of targeted therapy in cancer treatment in the era of precision medicine and immuno-oncology. It also helps to understand that the meaning or value of biomarker(s) and matched targeted therapy changes along with expansion of knowledge and advance of methodology, and constant efforts have to be made in evaluating the meaning and clinical value during the development and after the establishment of biomarkers or the approval of matched targeted therapies, which might be more complicated by the advent of new therapeutic agents and new diagnostic methods.

## Introduction

Targeted cancer therapy has already come to clinical practice and is one of the standards of care for patients with advanced or metastatic cancer. The National Cancer Institute defined it as drugs or other substances that block the growth and spread of cancer by interfering with specific molecules that are involved in the growth, progression and spread of cancer. This kind of specific molecules is called ‘molecular target’. Therefore, it is sometimes interchangeably used with ‘molecularly targeted therapies’ or ‘precision medicine’ or similar names. Compared with the standard cytotoxic chemotherapy, it is known to act on specific molecular targets in tumours, which translates into higher response rates and longer survival outcomes with better safety profile.

Identification of molecular targets, one of the predictive biomarkers, is very important for matched targeted therapy. From experiences in epidermal growth factor receptor tyrosine kinase inhibitor (EGFR TKI) therapy, targeted cancer therapy might be detrimental when given to patients without corresponding molecular targets or without identification of the target.^1^ On the contrary, even if molecular targets are identified but matched targeted therapy is absent or not given, survival outcomes might not differ between those without molecular targets and those without molecular targeted therapy.^2^ The identification of molecular targets or biomarkers is important as much as the administration of matched targeted therapy. In other words, targeted therapy is the standard of care for patients with molecular target(s), while cytotoxic chemotherapy is still a standard therapy for those without the targets, and immuno-oncology therapy can be a standard for some subsets of them.

There still remain many huddles to be overcome in successfully implementing targeted therapy in clinical practice, as well as in developing new targeted therapies. We will address some issues regarding molecular targets, especially predictive biomarkers, in relation to matched targeted therapies, hoping to provide better understanding and application of targeted therapy in cancer treatment in the era of precision medicine and immuno-oncology.

## Issues related to acquisition and evaluation of tissue

The first step of a targeted therapy starts with adequate and appropriate tumour tissues because the accuracy of molecular tests is affected by both the quality and the quantity of tumour tissues obtained, placing great emphasis on the importance of collecting and processing the samples. Recently, as a less or minimally invasive method when sampling tumour tissue is favoured, pathological diagnosis and classification and even molecular tests should be made in smaller biopsies and cytology specimens. On the contrary, as the number of biomarkers for testing is also increasing, smaller biopsies or cytology specimens make us face more difficult problems, preventing us from undergoing some or all of molecular tests needed, or requiring us to do additional or repeated biopsy.

Greater efforts should be made to educate physicians performing biopsies about the importance of obtaining a sufficient amount of tumour tissues. For example, molecular testing of lung adenocarcinoma is usually asked on small biopsy specimens obtained by either core needle biopsy (CNB) or fine-needle aspiration (FNA) biopsy. Although CNB yielded larger tumour tissues, it also increased the risk of serious bleeding or pneumothorax, causing reluctance among physicians.^3^ However, in a study regarding molecular testing, CNB specimens provided a significantly higher number of samples sufficient for molecular testing than did FNA specimens (67% vs 46%, p=0.007).^4^ Therefore, radiologists performing CT-guided lung biopsies are encouraged to use CNB than FNA, which can be helped in more formal and structured ways by shared protocols or existing guidelines.^5^ The success rate for CNBs improved from 72% to 92% after the guidelines were published jointly by the American Thoracic Society, the European Respiratory Society and the International Association for the Study of Lung Cancer.^6^ The relative lack of physicians’ knowledge and consensus on molecular testing might be an obstacle in implementing optimal process, but can also be compensated or overcome by regular multidisciplinary meetings or molecular tumour board.^7^ Developing general molecular testing policies and procedures at the institutional level based on available guidelines is also more helpful because it might improve workflow efficiency and testing performance.^8^ Guidelines are also meaningful in processing and storing tumour tissues, as well as in acquiring one. For instance, the National Comprehensive Cancer Network guideline recommends that efforts should be undertaken to minimise block reorientation and the number of tests before molecular testing. To do this, a limited number of immunohistochemistry (IHC) stains, which might suffice for most diagnostic problems, should be done or reserved only for cases in which the defining morphological criteria are absent.^9^ However, it might not be easily determined or established by each pathologist or at a pathology laboratory or department level. Therefore each institution had better have their own standard operating procedures related to performing biopsy, processing or handling tumour tissues, interpreting data, reporting the tests and making treatment decisions with consensus and standards, which could apply to all physicians and persons. The established institutional policies or precedures might involve in either some or all types of tumors.

## Issues related to methodology

Besides logistic issues, there are methodology-related issues which also closely relate to tissue issues. Adopting a more sensitive method is very important to identify molecular targets accurately. It may also be another way to preserve tumour tissues because it might decrease the minimum requirement of testing materials. Regarding genetic sequencing, Sanger sequencing is the first-generation sequencing and usually requires the greatest degree of tumour enrichment. Therefore, it is not appropriate for detection of genetic aberrations in specimens with a tumour portion of less than 25%–30%. Real-time PCR can be used with higher sensitivity for specific mutations, but it can assess only those specific mutations, limiting us from adding or expanding molecular tests. In this regard, next-generation sequencing (NGS) might be an option. The sensitivity of NGS is known to be higher than Sanger sequencing; for example, the sensitivity of NGS to detect mutant allele is at a mutant allele frequency of 2%~10% compared with that of Sanger sequencing which is at a mutant allele frequency of 15%~25%.^10^ In addition, NGS has significant benefits for clinical screening capability as well as biomarker discovery owing to multiple target screening at one time, or massive parallel screening as well as quantitative assay. NGS is not a single method and can be used in different ways based on input materials and selection of targets: whole genome sequencing for DNA, whole exome sequencing for DNA, whole transcriptome sequencing for RNA, and target sequencing for DNA or RNA. In this regard, NGS performance on biopsies or cytology specimens depends on the targets selected for sequencing and analysis. Its clinical significance is affected by its ability to simultaneously interrogate the establishing and/or emerging targets, and to rapidly and accurately direct patients to matched targeted therapies. So far, only a limited set of biomarkers are routinely used in screening for matched targeted therapies. The sensitivity for the specific biomarkers is balanced with the availability of the matched targeted therapies. However, NGS can also interrogate large numbers of patients and screen vast portions of the genome, which might help us to discover a new biomarker and to identify new meanings of the emerging or establishing biomarker in the context of a panel of biomarkers. NGS might also help to accelerate the development of new targeted therapies, when paired with clinical trials. However, NGS also has barriers, including the upfront cost of implementation, difficulty of standardisation and validation, and dependence or lack of bioinformatics expertise, most of which are related to the fact that it is a very rapidly evolving technology with multiple platforms and complex workflows. Advances in understanding tumour biology and discovery of new effective treatments complicate the situation. Therefore, the sensitivity required should be balanced with the availability of matched targeted therapies and the purpose of methodology adopted. Nonetheless, the benefits of NGS will increase its use in pathology laboratories and clinical practice. Before adopting a much more sensitive method, it should be kept in mind that the pathologist’s simultaneous evaluation of the morphology of tumour specimens is very important to ensure avoidance of useless, but expensive molecular testing, and traditional approaches including fluorescence in situ hybridisation or IHC can be used complementarily.^11^

Liquid biopsy or using blood or plasma samples is an alternative to tissue biopsy or can even replace tissue biopsy especially in cases when biopsy or cytology samples are absent or not available. Circulating tumour cells (CTCs) are found in frequencies in the order of 1~10 CTC/mL of whole blood in patients with metastatic disease, while circulating tumour cell-derived DNA (ctDNA) are found at up to 3.3% of tumour DNA, which may enter the blood every day.^12 13^ The more sensitive methods mentioned above, such as droplet digital PCR or NGS, decrease the minimum requirement of materials, which can make liquid biopsy feasible or useful in clinical practice. As well as the same dilemmas that the methods themselves face, liquid biopsy has a unique problem in that the concordance rates of the mutation status between the plasma and tumour samples are variable from 33% to 87%. There are errors in NGS depending on the platforms, with rates between 0.1% and 1%. A mutation with an allele frequency below a threshold cannot be differentiated from the background noise, and therefore protocols should be developed to reduce the error rate, for example, by incorporation of specific molecular targets identified prior to PCR amplification. However, the trade-off for high sequencing coverage is complete genomic landscape or even gene panels. The same problem occurs when choosing different NGS methods. Actually, whole genome sequencing coverage is on average below 100-fold, whereas targeted sequencing reaches a coverage of greater than 1000-fold, which directly correlates with the capability of target identification with low mutation burden. Fortunately, the novel NGS methods improve the sensitivity of NGS by performing highly targeted hybrid capture, high-throughput deep sequencing and utilisation of bioinformatics tools in order to remove artefacts and discover rare mutations, which make us overcome the problem of the very limited amount of DNA from CTC or ctDNA. Thus, owing to advancement in NGS, liquid biopsy might become a good alternative because it can give us information on specific molecular targets and on the genetic landscape of tumour heterogeneity. It might also provide insight into tumour dynamics, such as earlier prediction of response or detection of recurrence. So far, liquid biopsy still needs more technological advances for implementation and utilisation in routine practice, but it will complement or replace tissue biopsy because it is less invasive and more convenient.

As well as more sensitive methodologies, a kind of multiplex testing or parallel sequencing is needed. A variety of methods might be needed as the number of biomarkers to be tested is increasing due to the increment in the available matched targeted therapy.^2^ However, they are traded off against loss of tumour tissue as well as turnaround time and cost. In this regard, multiplex or massive parallel sequencing is more appropriate. However, determining the number of genes, which should be included, depends on the intended use or clinical consequence, and is not easy. Only core genes can be included with regard to their therapeutic relevance, but they might be insufficient as new genetic aberrations might have relevance. A larger gene set can be included for both clinical relevance and research purpose. Actually whole genome sequencing or exome sequencing can identify a wide range of genomic alterations, including known disease-associated genetic changes, but also novel variants, which might be suitable for research rather than clinical application or matched targeted therapies because of the trade-off mentioned above. For instance, The Cancer Genome Atlas Research Network used whole exome sequencing for lung adenocarcinoma and identified mutant genes, the clinical meanings of which are already well known in some but yet unknown or absent in others: *TP53* mutations (46%), *KRAS* (33%), *EGFR* (14%), *BRAF* (10%), *STK11* (17%), *KEAP1* (17%), *NF1* (11%), *SETD2* (9%), *RBM10* (8%), *ARID1A* (7%), *SMARCA4* (6%), *PIK3CA* (7%), *MET* (7%), *RB1* (4%), *U2AF1* (3%) *CDKN2A* (4%) and *RIT1* (2%), and very rare mutations as well. Some genetic aberrations needed a combination of DNA with mRNA sequencing, such as fusions or translocations involving *ALK*, *ROS1* and *RET*.^14^ On the contrary, targeted sequencing can focus on known or key genomic alterations in a small fraction of the genome or exome. It is more practical in the clinical setting and is the most frequently used type of NGS for molecular diagnostic testing because of its lower cost, easier bioinformatics interpretation, faster sample throughput and lower data storage requirement, as well as a higher coverage of sequencing with higher sensitivity and accuracy of detecting mutations. In addition, targeted sequencing-based pan-cancer panels in comparison with disease-specific panels might be more attractive in that they allow batching of samples across multiple indications, and save cost, labour and turnaround times. Therefore, when planning or designing a multiplex panel, the laboratory should define first its intended use, including what type of information will be needed or gathered, which influences the design, standardisation, validation, and quality control and assurance.^15 16^

## Rediscovery of meanings of biomarker

To date, we usually try to identify a molecular target or oncogenic driver for matched targeted therapy based on the efficacy evaluated and reported in prior clinical trials using specific molecular tests, which usually identify the presence of the target only. However, many resistance mechanisms were discovered and if possible hoped to be identified simultaneously. For instance, the efficacy of EGFR TKI therapy for *EGFR*-mutant patients might be affected by the presence of *TP53* comutations, which are not identified by a currently approved method, such as the cobas EGFR Mutation Test, but can be identified more by a more sensitive NGS method. In one study, the positive rate of *TP53* mutation was reportedly 48% by NGS but 8% by non-NGS.^17^ In a study at Memorial Sloan Kettering Cancer Center, *TP53* comutation was found in 62% of *EGFR*-mutant patients, and when treated with erlotinib patients with wild-type *TP53* had longer progression-free survival (PFS) than those with *TP53* comutation (median PFS: not reached vs 16 months, HR 2.7, p=0.017).^18^ In a study at Princess Margaret Cancer Centre, dual *EGFR* and *TP53* mutation was found in 41%, and those with wild-type *TP53* had a slightly higher response rate (52% vs 66%, p=0.46) and longer PFS (HR 1.82 (95% CI 1.03 to 3.22), p=0.039) to first-generation EGFR TKIs.^19^ In a study at Dana-Farber Cancer Institute, patients with co-*TP53* mutation survived shorter (median survival time: with *TP53* mutation vs without *TP53* mutation, 2.9 years vs not reached, p=0.02).^17^ The majority of tumour *TP53* mutations are known missense mutations, resulting in the accumulation of dysfunctional p53 protein, and these mutants often have oncogenic gain-of-function and sometimes exacerbate the malignant properties of tumour cells.^20^ Although there is no matched targeted therapy for *TP53* mutation yet, *TP53* mutation should be included in target sequencing-based pan-cancer panels. And each targeted therapy should be evaluated again in the context of the gene panel rather than a single gene itself even after the approval, which might guide a new therapeutic approach or lead to combination trials.

The efficacy of targeted therapy should be re-evaluated according to the methods used to identify the corresponding targets. Differences in the detection limit of testing platforms might have an impact on their test results, leading to different treatment strategies and eventually different clinical outcomes. The quantity or burden, not the presence or quality, of the molecular marker might also influence the outcome. In this regard, *T790M* comutation in *EGFR*-mutant patients might be representative. In one study, de novo *T790M* mutation was identified in 5% by Sanger sequencing, but in 41% by matrix-assisted laser desorption ionisation-time of mass spectrometry (MALDI-TOF MS). Of more interest, the response rate to first-generation EGFR TKIs of *T790M*-postive patients identified by MALDI-TOF was 56%, which is unexpectedly higher than that of *T790M*-positive patients identified by Sanger sequencing in the literature.^21^ In another study using the same method, the frequency of *T790M* mutation was 25% and the response rate to first-generation EGFR TKI in *T790M-*positive patients was 71%, which was comparable with 83.9% in *T790M-*negative patients. Of note, according to mutation burden reflecting subclones, the median progression-free survival and overall survival of low *T790M* patients were 6.7 months and 18.7 months, respectively, while those of high *T790M* patients were 2.4 months and 9.1 months, respectively. *T790M*-negative patients showed better survival outcome with a median progression-free and overall survival of 11.5 months and 26.5 months, respectively.^22^ Similar findings or different findings were observed in other studies according to the methods used.^23–25^ If the targeted therapy is matched by a new sensitive method, such as NGS, its efficacy should be re-evaluated in the context of the NGS or multiplex testing. If liquid biopsy is adopted for matching targeted therapy, its efficacy should also be evaluated again like in the context of different methodologies, considering different sensitivities and tumour burden.^26^ In order to use NGS or other multiplex platforms instead of available companion or complementary diagnostics, established or available targeted therapy should be re-evaluated in prospective clinical trials or at least through comparative effective research. In prospective clinical trials of an emerging or developing targeted therapy, using NGS or other multiple platforms should be considered emerging or developing targeted therapy even though the efficacy based on NGS is not the primary endpoint. The value of biomarkers and matched targeted therapy should be evaluated in terms of cost-effectiveness as well. However, the cost-effectiveness analysis might need control arms or marker-negative cohorts. Including marker-negative or wild-type patients may enhance the enthusiasm of the study and will sometimes give the patients access to a potentially beneficial experimental treatment. But it may not be feasible or reasonable or even unethical. What is worse is that the population might shift to another treatment during the trial as a new biomarker is identified or a new therapeutic approach is available. Therefore, to overcome this kind of problems, comparative effective research, which addresses the relative effectiveness comparing two or more tests or treatment or even policies of interest, should be considered or encouraged, especially in evaluating NGS-based approaches. Those studies can be randomised trials, but they deal with heterogeneous populations or real-world populations by mainly collecting already existing NGS data from larger healthcare databases.

Besides the issues mentioned above, the frequency of biomarkers is usually very low or rare,^14^ raising two problems in developing new targeted therapies: one relates to difficulty in identifying rare biomarkers for the targeted therapy, and the other is difficulty in developing the targeted therapy partly due to unclear market forecast with market profitability and market share. The former can be overcome partly by increased use of NGS multiplex testing in many laboratories. The latter could be overcome by new clinical trial designs such as umbrella or basket trial. New clinical trials using a kind of master protocol, as well as NGS-based methods in the context of basket, umbrella or platform trials, could be a good way to accelerate clinical development with increased efficiency and reduced cost, and also a better way to evaluate the value of established biomarkers and matched targeted therapy.

## Conclusion

The meaning or value of biomarker(s) and matched targeted therapy is not fixed. It might change as knowledge expands and methodology advances. It might also change as a new therapy with different mechanisms of action comes up. Immuno-oncology or immune checkpoint inhibitor therapy is a recent typical example. For *BRAF* mutation-positive patients with melanoma, *BRAF* inhibitors such as vemurafenib or dabrafenib were regarded as the standard therapy, but in a short time the combination of *BRAF* inhibitor and *MEK* inhibitor became the standard therapy. However, now a single anti- programmed cell death protein 1 (PD-1)/ programmed death-ligand 1 (PD-L1) immune checkpoint inhibitor takes over the position. Sooner or later combination of immune checkpoint inhibitors or anti-PD-1/PD-L1 inhibitor and anti-CTLA4 inhibitor might replace the single-agent therapy. Therefore, constant efforts have to be made in evaluating the meaning or role of biomarkers and matched targeted therapies during the development and even after the establishment or the approval.

## References

[1] Gridelli C, Ciardiello F, Gallo C, et al First-line erlotinib followed by second-line cisplatin-gemcitabine chemotherapy in advanced non-small-cell lung cancer: the TORCH randomized trial. J Clin Oncol 2012;30:3002–11. 10.1200/JCO.2011.41.2056 22778317

[2] Kris MG, Johnson BE, Berry LD, et al Using multiplexed assays of oncogenic drivers in lung cancers to select targeted drugs. JAMA 2014;311:1998–2006. 10.1001/jama.2014.3741 24846037 PMC4163053

[3] Geraghty PR, Kee ST, McFarlane G, et al CT-guided transthoracic needle aspiration biopsy of pulmonary nodules: needle size and pneumothorax rate. Radiology 2003;229:475–81. 10.1148/radiol.2291020499 14595149

[4] Schneider F, Smith MA, Lane MC, et al Adequacy of core needle biopsy specimens and fine-needle aspirates for molecular testing of lung adenocarcinomas. Am J Clin Pathol 2015;143:193–200. 10.1309/AJCPMY8UI7WSFSYY 25596245

[5] Lindeman NI, Cagle PT, Beasley MB, et al Molecular testing guideline for selection of lung cancer patients for EGFR and ALK tyrosine kinase inhibitors: guideline from the College of American Pathologists, International Association for the Study of Lung Cancer, and Association for Molecular Pathology. J Thorac Oncol 2013;8:823–59. 10.1097/JTO.0b013e318290868f 23552377 PMC4159960

[6] Ferretti GR, Busser B, de Fraipont F, et al Adequacy of CT-guided biopsies with histomolecular subtyping of pulmonary adenocarcinomas: influence of ATS/ERS/IASLC guidelines. Lung Cancer 2013;82:69–75. 10.1016/j.lungcan.2013.07.010 23927885

[7] van der Velden DL, van Herpen CML, van Laarhoven HWM, et al Molecular tumor boards: current practice and future needs Ann Oncol. 2017;28:3070–5. 10.1093/annonc/mdx528 29045504

[8] Cree IA, Deans Z, Ligtenberg MJL, et al Guidance for laboratories performing molecular pathology for cancer patients. J Clin Pathol 2014;67:923–31. 10.1136/jclinpath-2014-202404 25012948 PMC4215286

[9] National Comprehensive Cancer Network. Non-small cell lung cancer Cancer (Version 3, 2018). http://www.nccn.org/professionals/physician_gls/pdf/nscl.pdf (accessed 25 Mar 2018).

[10] Portier BP, Kanagal-Shamanna R, Luthra R, et al Quantitative assessment of mutant allele burden in solid tumors by semiconductor-based next-generation sequencing. Am J Clin Pathol 2014;141:559–72. 10.1309/AJCP1JUGQMW7ZNTL 24619758

[11] Cree IA, Deans Z, Ligtenberg MJ, et al European Society of Pathology Task Force on Quality Assurance in Molecular Pathology; Royal College of Pathologists. Guidance for laboratories performing molecular pathology for cancer patients. J Clin Pathol 2014;67:923–31. 25012948 10.1136/jclinpath-2014-202404 PMC4215286

[12] Schwarzenbach H, Hoon DS, Pantel K Cell-free nucleic acids as biomarkers in cancer patients. Nat Rev Cancer 2011;11:426–37. 10.1038/nrc3066 21562580

[13] Crowley E, Di Nicolantonio F, Loupakis F, et al Liquid biopsy: monitoring cancer-genetics in the blood. Nat Rev Clin Oncol 2013;10:472–84. 10.1038/nrclinonc.2013.110 23836314

[14] Cancer Genome Atlas Research Network. Comprehensive molecular profiling of lung adenocarcinoma. Nature 2014;511:543–50. 10.1038/nature13385 25079552 PMC4231481

[15] Jennings LJ, Arcila ME, Corless C, et al Guidelines for Validation of Next-Generation Sequencing-Based Oncology Panels: A Joint Consensus Recommendation of the Association for Molecular Pathology and College of American Pathologists. J Mol Diagn 2017;19:341–65. 10.1016/j.jmoldx.2017.01.011 28341590 PMC6941185

[16] Roy S, Coldren C, Karunamurthy A, et al Standards and Guidelines for Validating Next-Generation Sequencing Bioinformatics Pipelines: A Joint Recommendation of the Association for Molecular Pathology and the College of American Pathologists. J Mol Diagn 2018;20:4–27. 10.1016/j.jmoldx.2017.11.003 29154853

[17] Johnson BE The impact of next generation sequencing on the outcomes of patients with lung cancer. Paris, France: Plenary presentation O06.1 at: WIN 2016 Symposium, 2016.

[18] Ha Y, Jordan E, Mi A, et al Concurrent genetic alterations identified by next-generation sequencing in pre-treatment, metastatic EGFR-mutant lung cancers. J Clin Oncol 2016;34:abstr 9053.

[19] Labbe C, Korpanty G, Tomasini P, et al Prognostic and predictive effects of TP53 mutation in patients with EGFR-mutated non-small cell lung cancer (NSCLC). J Clin Oncol 2016;34:abstr 11585. 10.1016/j.lungcan.2017.06.014 28838393

[20] Rivlin N, Koifman G, Rotter V p53 orchestrates between normal differentiation and cancer. Semin Cancer Biol 2015;32:10–17. 10.1016/j.semcancer.2013.12.006 24406212

[21] Su KY, Chen HY, Li KC, et al Pretreatment epidermal growth factor receptor (EGFR) T790M mutation predicts shorter EGFR tyrosine kinase inhibitor response duration in patients with non-small-cell lung cancer. J Clin Oncol 2012;30:433–40. 10.1200/JCO.2011.38.3224 22215752

[22] Lee Y, Lee GK, Lee YS, et al Clinical outcome according to the level of preexisting epidermal growth factor receptor T790M mutation in patients with lung cancer harboring sensitive epidermal growth factor receptor mutations. Cancer 2014;120:2090–8. 10.1002/cncr.28711 24737599

[23] Fujita Y, Suda K, Kimura H, et al Highly sensitive detection of EGFR T790M mutation using colony hybridization predicts favorable prognosis of patients with lung cancer harboring activating EGFR mutation. J Thorac Oncol 2012;7:1640–4. 10.1097/JTO.0b013e3182653d7f 22899358

[24] Costa C, Molina MA, Drozdowskyj A, et al The impact of EGFR T790M mutations and BIM mRNA expression on outcome in patients with EGFR-mutant NSCLC treated with erlotinib or chemotherapy in the randomized phase III EURTAC trial. Clin Cancer Res 2014;20:2001–10. 10.1158/1078-0432.CCR-13-2233 24493829

[25] Yang JC, Sequist LV, Geater SL, et al Clinical activity of afatinib in patients with advanced non-small-cell lung cancer harbouring uncommon EGFR mutations: a combined post-hoc analysis of LUX-Lung 2, LUX-Lung 3, and LUX-Lung 6. Lancet Oncol 2015;16:830–8. 10.1016/S1470-2045(15)00026-1 26051236

[26] Oxnard GR, Thress KS, Alden RS, et al Association Between Plasma Genotyping and Outcomes of Treatment With Osimertinib (AZD9291) in Advanced Non-Small-Cell Lung Cancer. J Clin Oncol 2016;34:3375–82. 10.1200/JCO.2016.66.7162 27354477 PMC5035123

